# Supplementary material for: Ploidy inference from single-cell data: application to human and mouse cell atlases
Source: Genetics. 2024 Apr 23;227(2):iyae061. doi: 10.1093/genetics/iyae061 (PMC11151930; doi:10.1093/genetics/iyae061)

Figure S1

The effect of next-generation sequencing depth on the accuracy of ploidy inference algorithms. In comparison to the simulation in Figure 2, a subsample of 50% or 25% of next-generation sequencing reads (upper panel and lower panel, respectively) was used as input. See the legend of Figure 2 for further details.

Sub-sampling 50% of reads.

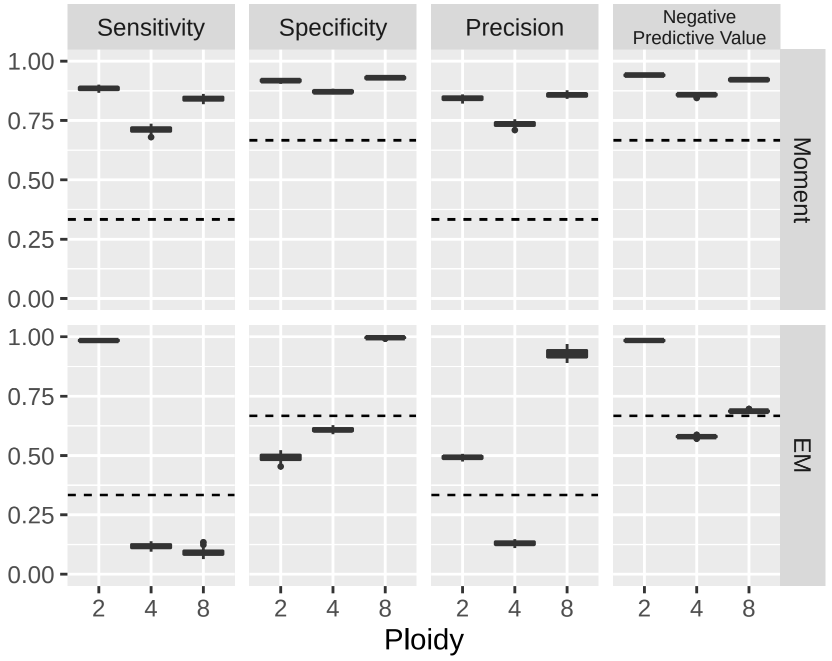

Sub-sampling 25% of reads.

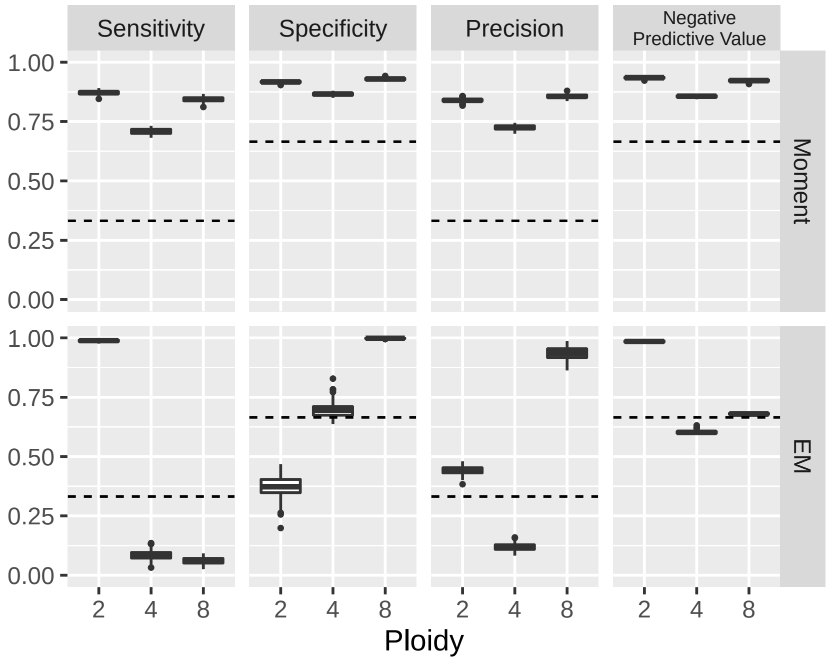

Supplement: iyae061_Supplementary_Data [file iyae061_supplementary_data.zip › Supplementary_Figure_S1_GENETICS-2024-306952.pdf]
